# Coronary accordion effect mimicking coronary dissection in a 41-year-old male during angiography procedure an interesting case report

**DOI:** 10.1016/j.radcr.2024.06.083

**Published:** 2024-08-07

**Authors:** Said A. Ahmed, Feyza Aksu, Mohamed O. Hassan

**Affiliations:** Cardiology Department, Mogadishu Somali-Turkish Training and Research Hospital, Mogadishu, Somalia

**Keywords:** Coronary angiography, Coronary artery disease, Electrocardiography, Coronary vessels

## Abstract

The accordion phenomenon is a rare pseudo-complication observed during percutaneous coronary intervention (PCI), which can mimic coronary dissection, spasm, or thrombus formation. Here we present a patient with inferior ST-segment elevation myocardial infarction (STEMI), who developed multiple pseudo lesions in the tortuous right coronary artery (RCA) during PCI. An emergency coronary angiography was performed, but unfortunately, the lesion developed into an accordion-like shape in the middle segment of the right coronary artery (RCA), which looked like a coronary dissection. Despite attempts to resolve the abnormality with intracoronary nitrates, the accordion effect persisted, leading to a drop in blood pressure. Subsequent administration of isotonic solution and additional intracoronary nitrates eventually alleviated the dissection pattern. Due to the small diameter of the posterior descending artery, balloon angioplasty was performed to complete the procedure successfully. The patient was discharged 2 days later with a prescribed regimen of aspirin, prasugrel, atorvastatin, and carvedilol. Follow-up after 1 week indicated the patient's well-being, with no reported complaints.

## Introduction

The accordion phenomenon is a rare pseudo-complication observed during percutaneous coronary intervention (PCI), which can mimic coronary dissection, spasm, or thrombus formation [1]. It is a guidewire-induced pseudo-lesion during the percutaneous coronary intervention (PCI) in tortuous vessels [2]. It describes the mechanical distortion of tortuous arteries caused by any material that stretches the artery, leading to invagination or shortening of the vessel wall, resulting in an accordion-like appearance [3]. Here we present a patient with ST-segment elevation myocardial infarction (STEMI), who developed multiple pseudo lesions in the tortuous right coronary artery (RCA) during PCI.

## Case presentation

**Fig. 1:**
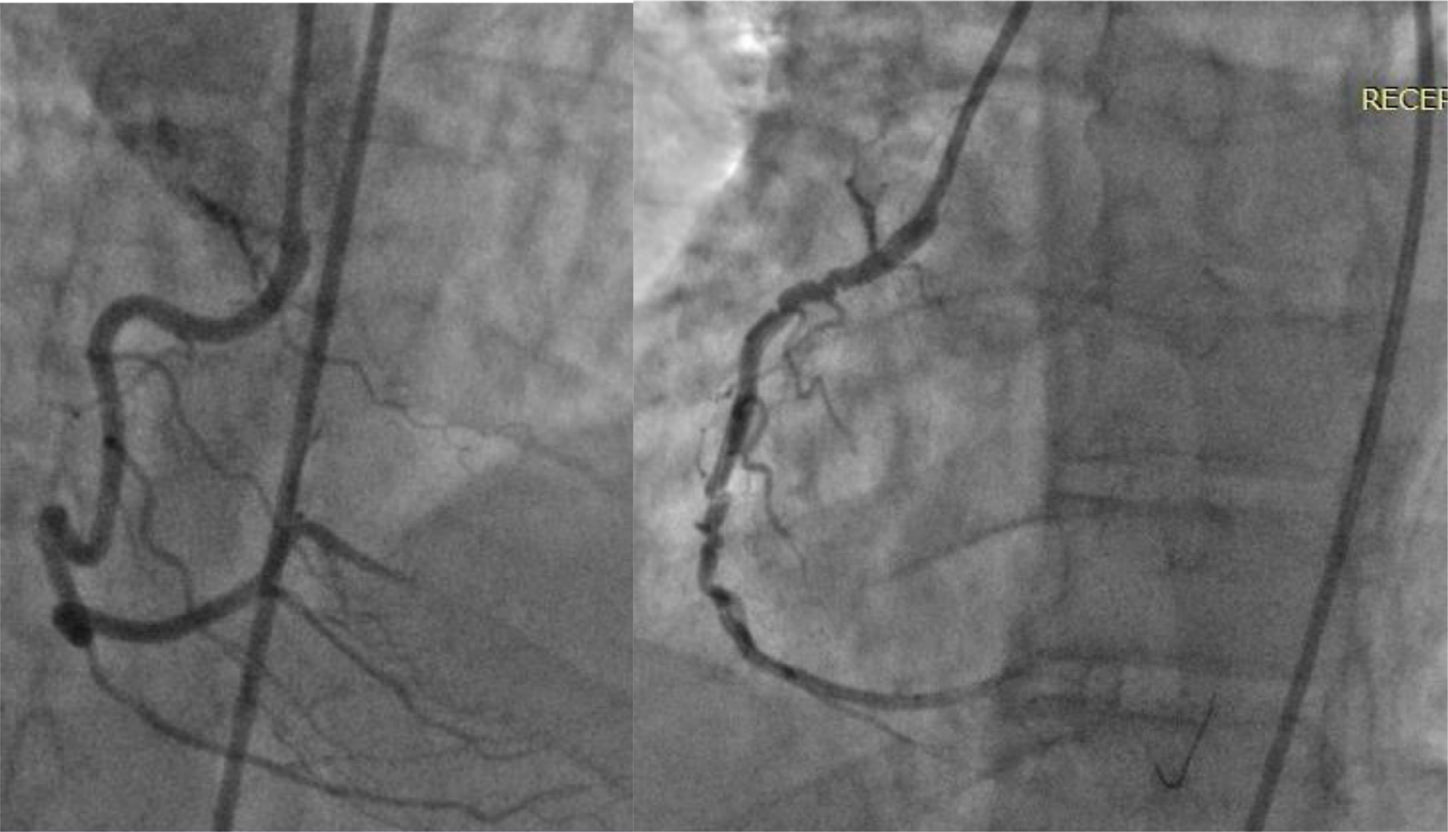
(A) Right coronary artery showing tortuous with distal PDA. (B) RCA showing pseduolesions mimimkg dissection with loss of follow.

**Fig. 2:**
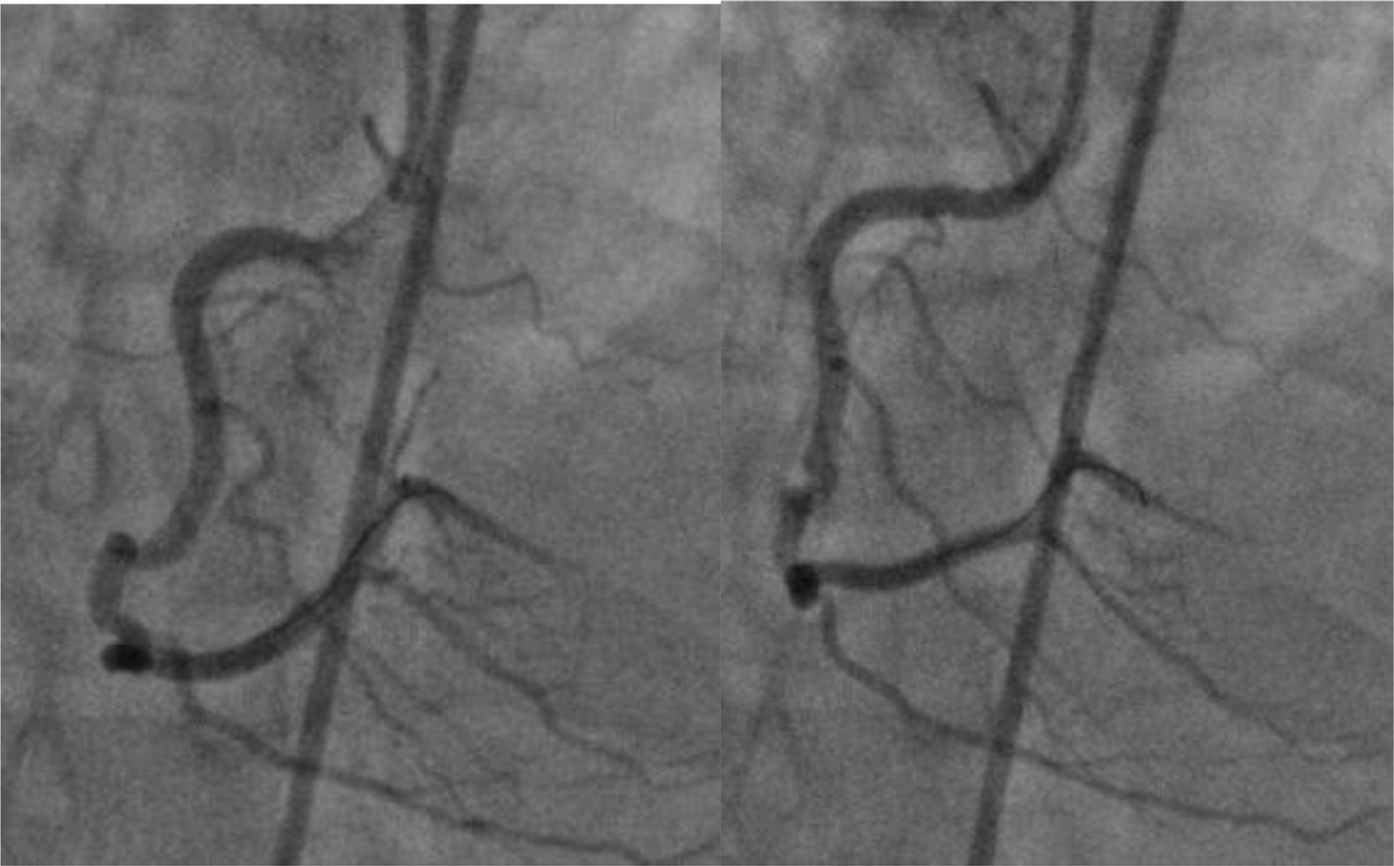
Panel A Showing partial resolution of psedolesion after catheter disengagement and IC nitrate. Panel B Re-engagment and final imaging.

A 41-year-old male with a history of intermittent chest discomfort and hypertension, presented to our cardiology department following an episode of severe chest pain. The pain was described as substernal, radiating to the left arm, and associated with shortness of breath. No previous cardiac events or interventions were reported. Upon arrival, the patient was in distress but hemodynamically stable. Blood pressure was 140/90 mmHg, and heart rate was 95 beats per minute. Physical examination revealed no signs of heart failure, and lung fields were clear on auscultation. Electrocardiogram (ECG) showed ST-segment elevation in leads II, III, and AVF. Troponin Levels were elevated, confirming myocardial injury. Emergency coronary angiography was performed to assess the extent of coronary involvement. RCA was engaged with JR/6F and the lesion was crossed with floppy wire. Suddenly the catheter aspirated deeply into RCA and the angiogram displayed an unusual accordion-like appearance in the mid-segment of the right coronary artery (RCA) artery mimicking the characteristics of a coronary dissection Fig. 1A and B. After several nitrate infusions the patient figures. After catheter disengagement and several intracoronary nitrates, the accordion effect persisted, and the patient's blood pressure began to drop. A bolus isotonic solution was started, and after 5 minutes, the blood pressure improved slightly, so another IC nitrate was administered. Fortunately, the accordion effect subsided (2A&B). Because the diameter of the posterior descending artery was less than 2.5 mm, the lesion was ballooned, and the procedure was completed without further complications. Two days later, the patient was released with aspirin 100 mg 1 × 1, prasugril 90 2 × 1, atorvastatin 40 mg 1 × 1, and carvedilol 6.25 mg 2 × 1. After one week of cardiology outpatient follow-up, the patient was doing well with no complaints (Fig. 2).

## Discussion

Myocardial infarction and cardiovascular disease are major global health concerns, especially in Somalia. Early diagnosis and timely management are essential to reducing complications related to myocardial necrosis [4]. Furthermore, complications such as the accordion effect during intervention must be understood in order to avoid unnecessary interventions. The highest incidence of this phenomenon is seen when highly tortuous arterial vessels are linearized with a stiff guidewire, and it can simply be reversed by withdrawing the mechanical device, causing the artery deformity [5]. Therefore Removing a stiff guidewire is a commonly used technique for diagnosing and treating accordion phenomenon . Prior to removing the guidewire, intravascular ultrasound imaging may be useful to rule out the presence of a dissection or thrombus [6]. Inexperienced operators may make unnecessary interventions like stenting of the artery from proximal to distal vessel. If not treated promptly, this phenomenon can lead to ischemia and hemodynamic compromise due to reduced or complete flow loss [7].

## Conclusion

This case emphasizes the importance of considering atypical coronary presentations during angiography procedures. Recognizing a coronary accordion effect is crucial to avoid unnecessary interventions associated with traditional dissections. Clinicians should remain vigilant and employ a multidisciplinary approach to ensure accurate diagnosis and appropriate management in such unique cases.

## Patient consent

Written informed consent for the publication of this case report was obtained from the patient.

## Author contribution

All authors contributed toward writing, analysis, drafting, and revising the paper and they gave final approval of the version to be published, and agree to be accountable for all aspects of the work.
